# Instagram Addiction in Italian Young Adults: The Role of Social Influence Processes, Meaningful Relationships and Fear of Missing Out

**DOI:** 10.3390/bs15121711

**Published:** 2025-12-10

**Authors:** Venusia Covelli, Alessandra Marelli, Marina Angela Visco, Pietro Crescenzo, Alessandra Bavagnoli

**Affiliations:** Department of Theoretical and Applied Sciences, e-Campus University, Via Isimbardi, 10-22060 Novedrate, Italy; marina.visco@uniecampus.it (M.A.V.); pietro.crescenzo@uniecampus.it (P.C.); alessandra.bavagnoli@uniecampus.it (A.B.)

**Keywords:** Instagram addiction, young adults, social influence processes, attachment, FoMO

## Abstract

Research on Instagram addiction (IA) has examined a range of psychological and socio-relational factors to explain the addiction, including personality traits, self-esteem, mental health, social approval, and fear of missing out (FoMO), among others. However, no study has integrated both social influence processes (subjective norms, group norms, and social identity) and meaningful relationships (attachment, dyadic, and friendship ties) with FoMO in relation to IA. This study examined the interplay among social influence processes, meaningful relationships, and FoMO on IA, as well as the moderating roles of subjective and group norms on the indirect effect of anxious attachment on IA via FoMO. The sample consisted of 180 Italian young adults (aged 18–30) who completed validated questionnaires on IA, social influence, relationships, and FoMO after providing consent. Social media use was also explored through an open-ended question. Results indicate that FoMO, social and group norms, and group identification significantly contribute to IA. Anxious attachment had a significant indirect effect on IA via FoMO, with subjective and group norms moderating this association. Qualitative analysis of open-ended responses enriched the understanding of young adults’ social media use. These findings highlight the importance of social influence, relationships, and FoMO in young adults’ Instagram engagement and suggest directions for addressing problematic use in this group.

## 1. Introduction

### 1.1. Social Media Usage, Psychological Effects, and Instagram Addiction

Social networks have become an integral part of daily life, with 62.3% of the global population using them, totaling 5.07 billion active users (Statista, 2024). Among these platforms, Instagram, WhatsApp, Facebook, WeChat and TikTok are the social networks most frequently used by the global population (Digital Global Overview Report, 2024), with Instagram alone boasting 2 billion monthly active users worldwide and 27 million in Italy, where it is especially popular among young people aged 18–29 (Statista, 2024; Soraci et al., 2022). Adolescents and young adults are the main users and are the most at risk for mental health disorders linked to excessive social media (Khalaf et al., 2023) and smartphone (Della Vedova et al., 2022; Wacks & Weinstein, 2021) use. A recent literature review (Koh et al., 2024) found that excessive and passive social media use among adults is often associated with increased depression, anxiety, negative mood, and feelings of loneliness. However, about one-third of the studies also report positive effects, with targeted and positive use contributing to improved perceived social support and psychological well-being (Koh et al., 2024).

Although Instagram has been popular since its launch in 2010, research on its problematic use and Instagram Addiction (IA) has only recently emerged (Kırcaburun & Griffiths, 2019; Pekpazar et al., 2021; Soraci et al., 2022). Problematic use occurs when frequent use becomes a habit, and individuals begin relying on the platform to manage stress or negative emotions. This can lead to signs of behavioral addiction, such as difficulties in other important areas of life due to social network use (Kircaburun & Griffiths, 2018; Griffiths, 2005). When this pattern intensifies—marked by an uncontrollable urge to use the platform, a constant desire to stay connected, and continued use despite serious negative consequences—it can be considered an addiction. However, it is important to note that, although these behaviors appear to be addictive, they are generally not recognized as disorders in major international diagnostic manuals, such as the DSM-5 (APA, 2013) and the ICD-11 (WHO, 2019; Soraci et al., 2022). In this study, we use the term “Instagram addiction” following other authors, such as Kircaburun and Griffiths (2018), who have employed the same terminology, and because Soraci et al. (2022) applied the Instagram Addiction Scale to measure Instagram-related addictive behaviors. The same observation was made by Della Vedova et al. regarding internet addiction, which similarly lacks recognition as a mental disorder in the DSM-5, whereas “gaming disorder” has been included in the ICD-11.

### 1.2. Instagram Addiction: Risk Factors, Correlates, and Moderators

IA may arise from a combination of biological, psychological, and social factors (Griffiths, 2005). It is often viewed as a complex condition that requires analysis from multiple disciplines (Kircaburun & Griffiths, 2018). Research so far has identified links between IA and excessive online activity, as well as personality traits, negative mood, depression, stress, anxiety, emotional exhaustion, poor mental health, and low life satisfaction (Fard et al., 2025; Herrero-Báguena et al., 2025; Ballarotto et al., 2021; Sanz-Blas et al., 2019; Yurdagül et al., 2021; Soraci et al., 2022; D’Souza & Hemamalini, 2018). Other studies have connected IA to body dissatisfaction related to sexualized female images (Guizzo et al., 2021), social anxiety, negative social comparisons, loneliness, and difficulties in real-life social relationships (Rogowska & Libera, 2022; Lopez & Polletta, 2021; Yurdagül et al., 2021). Associations with poor academic performance (Pekpazar et al., 2021), low self-esteem, poor sleep, bullying, fear of missing out, cyberbullying, and sexual dysfunction have also been observed (Longobardi et al., 2020; Soraci et al., 2022). Recent findings highlight that critical thinking can serve as a protective factor against social media addiction and IA: individuals with strong critical thinking skills tend to seek different sources of satisfaction and social connection, lowering their dependence on social media for approval and engagement. Conversely, escapism, social motives, and a desire to belong increase the risk (Fabio & Iaconis, 2024). Additionally, research exploring the links between IA, basic psychological needs, and psychological well-being suggests that fundamental needs—autonomy, competence, and relatedness—directly influence IA (Sharifi Fard et al., 2021). Concerning the age group in our study, some research focuses on adolescents (ages 11–19) (e.g., Longobardi et al., 2020; Yurdagül et al., 2021; Ballarotto et al., 2021), while others examine young adults, primarily college students aged 18–25 (Kircaburun & Griffiths, 2018; Sanz-Blas et al., 2019; Fabio & Iaconis, 2024; D’Souza & Hemamalini, 2018; Rogowska & Libera, 2022; Yesilyurt & Solpuk Turhan, 2020), adults over 18 (Lopez & Polletta, 2021; Fard et al., 2025; Herrero-Báguena et al., 2025; Soraci et al., 2022), or exclusively women (Guizzo et al., 2021).

### 1.3. Social Influence and Interpersonal Relationships in Problematic Social Media Use

Drawing from the literature on social media addiction and problematic use, researchers have demonstrated that, in addition to focusing on psychological factors such as personality traits, low self-esteem, and life satisfaction, a broader range of social factors has also been explored. These include social influence processes (subjective norms, group norms, and social identity) and important relational aspects (attachment, dyadic relationships, and friendship ties). Building on this perspective, studies grounded in Social Influence Processes (SIP) have examined group norms, subjective norms, and social identity, especially among young adults aged 18 to 28 (Marino et al., 2016; Zhou, 2011; Chen, 2018). Researchers have used social influence theory (Kelman, 1958) to explore how social factors can predict online behavior through compliance, internalization, and identification. According to this theory, subjective norms (which suggest that people act based on what others think to gain their approval), group norms (which imply that individuals accept group influence because their goals align with those of other group members), and social identity (which relates to a person’s sense of belonging and their desire to maintain meaningful or self-defining relationships with others in the group) can affect individual behaviors (Kelman, 1974; Shen et al., 2011; Zhou, 2011; Tajfel, 1979; Ellemers et al., 1999). This framework has been used to study social network addiction, especially on Facebook (Marino et al., 2016; de Oliveira et al., 2016), as well as online community participation (Zhou, 2011).

Alongside studies examining social influence processes, other research has also investigated relational and family variables, such as friendships, dyadic relationships, and attachment, to understand excessive use of social networks (Ballarotto et al., 2021; Satici et al., 2023). Studies examining the relationship between friendship quality and social media addiction, particularly in adolescents, indicate that social media addiction may diminish perceived closeness with both close friends and other contacts (Pouwels et al., 2021; Angelini et al., 2023; Zhang, 2023). However, the study of Hamad and Jaradat (2024) on university students indicates that two dimensions of friendship quality—trustworthy alliance and familiarity—predict social network addiction. Regarding couples’ satisfaction and excessive social media use, Goodman-Deane et al. (2016) report high overall satisfaction, with personal and voice communication positively correlated, while text and instant messaging were negatively correlated. Social media addiction is negatively correlated with commitment in romantic relationships (Abbasi, 2019; Abbasi et al., 2019), and addiction may lead to infidelity facilitated by social media, resulting in relationship breakdown and diminished satisfaction (Demircioğlu & Köse, 2021; Valenzuela et al., 2014; Satici et al., 2023). According to traditional attachment theory (Bowlby, 1969; Ainsworth, 1985; Bowlby, 1988), research has shown a positive correlation between insecure attachment (styles avoidant or ambivalent) and problematic social media use, particularly on Instagram and Facebook, among adolescents (Ballarotto et al., 2021) and young adults (with an average age 24) (Jimeno et al., 2022). Studies have also found that anxious attachment predicts social media addiction, as individuals seek security and attention online due to fears of abandonment (Baek et al., 2014; Blackwell et al., 2017; D’Arienzo et al., 2019; Sun & Zhang, 2021; Worsley et al., 2018).

### 1.4. Anxious Attachment, Fear of Missing Out, and Problematic Social Media Use

Evidence also shows that high levels of anxious attachment can lead to fear of missing out (FoMO) (Blackwell et al., 2017). Anxious attachment heightens fears related to social rejection and abandonment, causing individuals to be hypervigilant about missing important social interactions or experiences. This hypervigilance, driven by anxious attachment, explains why FoMO develops as individuals become increasingly concerned about being excluded from rewarding social experiences (Perazzini et al., 2023). Therefore, anxious attachment leads to FoMO by increasing sensitivity to social threats and the need for reassurance, making FoMO a direct consequence of the emotional dynamics of anxious attachment (Liu & Ma, 2019).

The FoMO concept is defined as “a pervasive apprehension that others might be having rewarding experiences from which one is absent” (Przybylski et al., 2013; Fioravanti et al., 2021). Research on FoMO has highlighted positive correlations with loneliness and depressive symptoms (Reer et al., 2019). FoMO is also linked to anxiety, with higher levels associated with increased anxiety severity (Blackwell et al., 2017; Elhai et al., 2019). Furthermore, FoMO negatively affects sleep habits (Scott & Woods, 2018) and is positively associated with increased alcohol consumption and related negative consequences among college students (Riordan et al., 2015). According to Self-Determination Theory (SDT), FoMO arises from unmet needs for competence, relatedness, and autonomy (Deci & Ryan, 1985). When these needs are not satisfied, maladaptive behaviors, like FoMO, can develop, especially in social media environments (Przybylski et al., 2013). Individuals with anxious attachment, due to unmet needs for affiliation or autonomy, are more likely to experience FoMO, which is linked to problematic social media use, including Facebook, Instagram, and WhatsApp (Błachnio & Przepiórka, 2018; Dhir et al., 2018). Recent studies have explored how FoMO mediates the relationship between anxious attachment and problematic social media use, confirming FoMO’s mediating role (Boustead & Flack, 2021; Liu & Ma, 2019). However, no studies have specifically examined the indirect effect of anxious attachment on IA through FoMO. Beyond individual attachment and FoMO, relational and family factors, together with SIP, have also been implicated in social network addiction (Chen, 2018; Marino et al., 2016). People with FoMO are influenced by social norms and the fear of missing out on others’ experiences (Li et al., 2023; Chai et al., 2018). While no direct evidence links SIP and attachment, some studies have examined how attachment to social groups (secure and insecure) relates to problematic behaviors such as gaming and internet use (Carvalho et al., 2023).

### 1.5. Study Aims and Hypothesis

Based on this evidence, the present study aims to deepen the understanding of IA among young adults by examining how social identity processes, individual attachment orientations, and FoMO interplay in IA. We propose that higher levels of subjective and group norms may interact with anxious attachment to foster FoMO, which in turn contributes to problematic social network use and IA. This integrated framework is supported by previous studies demonstrating that anxiety attachment, FoMO, and subjective norms are predictive of problem social network use, and how these factors together predict problematic social network use, particularly IA. Therefore, the present study had three aims. First, we explore Instagram use among young adults aged 18 to 30, including their personal narratives about social media engagement. As such, no formal hypothesis is proposed for this qualitative strand. Second, we investigate the interplay and underlying mechanisms among SIP (social identity, subjective norms, and group norms), meaningful relationships (attachment, dyadic, and friendship bonds), and FoMO in relation to IA. We hypothesize that each of these factors will be positively associated with problematic Instagram use (H1). Furthermore, we expect FoMO to operate as a psychological mechanism linking anxious attachment to IA, such that individuals with higher attachment anxiety will report higher FoMO, which in turn will predict more problematic use (H2). Third, we examine the moderating roles of subjective and group norms in the indirect effects of anxious attachment on IA via FoMO. We hypothesize that these norms will intensify the indirect impact, amplifying the mediation process when social expectations for Instagram engagement are particularly salient (H3). Using a mixed-methods approach, we aim to provide a comprehensive understanding of how social and relational factors jointly contribute to IA and to highlight the complex interactions underlying Instagram use among young adults.

## 2. Materials and Methods

### 2.1. Study Design and Data Collection

This is an exploratory, descriptive, mixed-methods study. We gathered both quantitative and qualitative data to gain an integrated view of Instagram use among a sample of Italian young adults (Guetterman et al., 2015). An online survey was conducted via Qualtrics from 1 April to 30 June 2023.

Recruitment was carried out through email invitations and social media platforms such as LinkedIn, Facebook, and WhatsApp, primarily targeting young adults residing in Italy. Although it was not possible to precisely track the number of contacts reached or impressions due to the use of multiple networks and snowball sampling, efforts were made to engage a diverse group within the target age range. No incentives were provided to minimize the risk of automated or insincere responses. The estimated average time to complete the questionnaire was 15 to 20 min.

### 2.2. Ethical Aspects

Ethical permission was obtained from the University’s Ethics Commission, in accordance with the Declaration of Helsinki (protocol code 06/2023; 5 August 2023). Participants were informed of the survey’s purpose and procedures. Informed consent was obtained before the survey began. Each participant agreed to take part in the study voluntarily and could decide to leave at any time.

### 2.3. Measures

*The use of social networks.* After answering some sociodemographic questions (e.g., gender, age, education level, marital status, region of residence, profession), participants were asked to provide some information about their social media use and Instagram. They reported which social networks they subscribe to besides Instagram and which one they use most often; whether they lose sleep by staying up late to use social media; how many hours they typically spend on Instagram daily; and what times of day they use it. Additionally, in an open-ended question, participants described their relationship with social media: “Now we ask you to tell us everything you can think of about you and social media, your relationship with social media (e.g., what role they play in your life and how they make you feel when you use them). You can write freely about anything that comes to your mind.”

*Instagram Addiction Scale (IAS).* The IAS-15 (Kircaburun & Griffiths, 2018; Italian version by Soraci et al., 2022) is used to evaluate participants’ level of Instagram addiction. It includes two subfactors, “social impact” and “compulsion”. It is a 6-point scale ranging from “never” to “always” with a possible score of 15 to 90 (α = 0.92). The higher the score, the greater the risk of IA.

*Social Influence Processes (SIP)*. SIP was assessed with a total of 15 items (3 items for “subjective norms”, 4 items for “group norms”, and 6 items for “social identity”) adapted to the Instagram context from earlier studies (Dholakia et al., 2004; Marino et al., 2016). They were scored on a 7-point scale from 1 = not at all to 7 = very much (Marino et al., 2016).

*Fear of Missing Out Scale* (FoMOs). A 10-item questionnaire used to assess FoMO (Przybylski et al., 2013; Italian version: Casale & Fioravanti, 2020). Items have a 5-point rating scale (1 = “not at all true of me” to 5 = “extremely true of me”). The total score ranges from 10 to 50, with a higher score indicating a higher level of FoMO. In this study, we created a combined index of the two subscales, “Fear” and “Control”, following previous studies (Benzi et al., 2024) (α = 0.94).

*Dyadic Adjustment Scale (DAS).* The Dyadic Adjustment Scale (DAS; Spanier, 1976; Italian validation: Gentili et al., 2002) is a 32-item self-report instrument designed to evaluate how couples feel about their relationship and how each partner feels about it. It is composed of 4 subscales: “dyadic satisfaction”, “dyadic cohesion”, “dyadic consensus”, and “affectional experience” (we used only the “dyadic satisfaction” subscale). This subscale contains 10 items about positive and negative communication between couples. An increase in the test score indicates a more positive dimension (α = 0.87).

*Adult Attachment Scale-Revised* (AAS-R). The Adult Attachment Scale-Revised (AAS-R; Troisi et al., 2022) was a revised version of the original Adult Attachment Scale (Collins, 1996) and assessed adult attachment. The AAS-R consisted of 18 items scored on a 5-point Likert-scale (1 = “not at all characteristics of me” to 5 = “very characteristic of me”). The three scales (“close”, “depend”, and “anxiety” showed sufficient internal consistency (α coefficients ranging from 0.69 to 0.75).

*Friendship Qualities Scale* (FQS). The Friendship Qualities Scale (FQS; Bukowski et al., 1994) is a multidimensional self-report measure of friendship quality. The Italian version is an equivalent 22-item self-report measure of friendship quality from adolescence to early adulthood (Ponti et al., 2010). The response options for each item are rated on a 5-point Likert scale (1 = “absolutely false” to 5 = “absolutely true”). The Italian version (Ponti et al., 2010) reveals, via confirmatory factor analysis, the same main qualitative dimensions and shows good reliability of the scales. In the present study, we used the “closeness” subscale, which showed good reliability (α = 0.79).

### 2.4. Data Analysis

Data were processed using SPSS software (version 21.0). To describe the socio-demographic characteristics of the sample, descriptive statistics were calculated. Pearson correlation coefficients were used to analyze the associations between variables.

We conducted a thematic analysis according to the procedure outlined by Braun and Clarke (2006) for the open question about their relationship with social media. The thematic analysis aimed to identify and describe the implicit and explicit themes emerging from participants’ open-ended responses. Two members of the research team (VC&AM) analyzed people’s responses and independently identified the themes that emerged.

**Figure 1:**
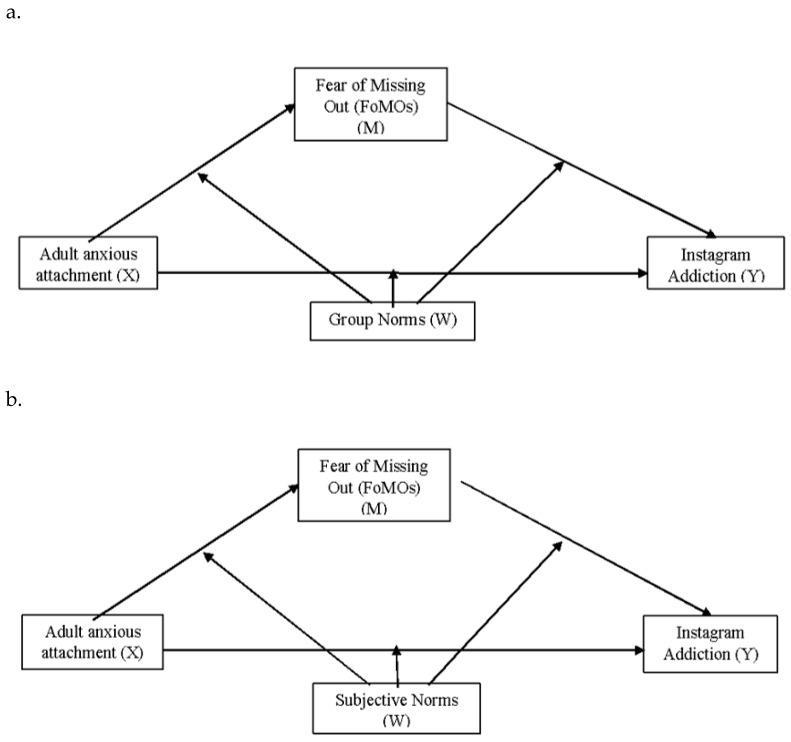
(**a**,**b**) The moderated mediation models.

The moderated mediation model was estimated using the bias-corrected bootstrap sampling approach (5000 samples), with the SPSS Process Macro (Hayes, 2017) and model 59, due to its special suitability for small samples (Hayes, 2012). This model (see Figure 1a,b) allowed for exploring whether subjective norms or group norms (W) moderated the indirect effects of anxious attachment (X) on IA (Y) through the mediation of FoMO (M).

## 3. Results

A total of 274 young adults initially participated in the survey and provided informed consent for their data to be processed. Of the 94 excluded from the analysis, 49 provided consent but did not continue with the compilation, 30 provided only socio-demographic information, and 15 stopped immediately after completing the Instagram Addiction Scale (IAS) without proceeding with the remaining questionnaires. For this reason, the total sample consisted of 180 young adults.

Table 1 shows the socio-demographic characteristics of the sample. Most participants were women (75.6%) with a mean age of 24.7 years (SD = 3.25), single (74.4%), and living with their family of origin (60%). They were also university students (28.9%), student workers (28.3%), and employed or self-employed (27.8%).

### 3.1. The Use of Instagram Among Young Adults

As reported in Table 2, most participants (97.8%) are registered on WhatsApp, the social media they use the most, and also use Instagram the most often (86.7%). Most of the sample (75.5%) reported occasionally losing hours of sleep due to late-night use of social networks (36.1%), with the evening being the most common time for use (39.4%). The average daily Instagram use for the sample is 2.35 h (SD = 1.33).

Moreover, 33.3% (n = 60) of participants responded to the open-ended question about their relationship with social networks. Based on the responses read and analyzed, we identified the six most common themes, which we grouped into three macro-themes. Overall, participants described their relationships with social networks by outlining the benefits and drawbacks of using them. On one hand, participants mentioned that it allows them to access a wide range of multimedia content (photos, videos, articles), stay connected and communicate, and even create job opportunities. On the other hand, they also noted how social networks can lead to digital addiction and social isolation. Table 3 presents the analysis results. Each identified theme is described below.

#### 3.1.1. Benefits of Using Social Network

*Relationship*. What emerges from this theme is that social networks make it possible to stay in contact with everyone, including people participants have not seen for a long time or distant family members. An excellent way to meet and get to know new people is through social networks. This can be on a personal or professional level. Some respondents said that meeting people and making new contacts was their primary use of social networks. Others, however, said they used social media to stay in touch with friends and family.

*Utility.* Participants’ responses indicated that social networks are the ideal place to find information. They reported that all you need to do is open social networks to learn what is new in the world or to get informed about a specific company, product, or service. Some participants mentioned using social networks to search for information on news, travel, cooking, products and brands, music, and videos. Others, however, stated that they primarily use social networks for work purposes, such as promoting their images, products, and brands, and increasing their followers.

*Filler for empty moments*. Social networks serve as fillers for empty, dull moments, thanks to the quantity and variety of content they offer. Participants reported using social networks particularly during work breaks, early in the morning, or in the evening before falling asleep.

#### 3.1.2. Costs of Using Social Networks

*Addiction.* Participants reported that social media can lead to digital addiction and feelings of isolation. Being constantly connected and overusing social media can disrupt traditional in-person relationships and reduce time spent offline.

*Privacy & Control.* Participants mentioned that another downside of using social networks was managing personal information. They said they use social networks to check on family, friends, and acquaintances, and to stay updated on their lives by viewing their posts and stories.

### 3.2. Interactions and Moderations Among SIP, Meaningful Relationships, and FoMO on IA

Before conducting mediation and moderation analyses, we examined correlations between variables to investigate the presence and direction of relationships, as a prerequisite for the subsequent models.

The correlational analyses (see Table 4) revealed significant links between the IAS and some of the investigated variables. Specifically, the IAS correlated with the three SIP explored in this study: subjective norms (r = 0.231, *p* < 0.01), group norms (r = 0.433, *p* < 0.01), and cognitive social identity (r = 0.172, *p* < 0.05). Moreover, the results showed high associations between the IAS and the FoMOs (r = 0.623, *p* < 0.01), the Security subscale in the FQS (r = −0.267, *p* < 0.01), the Anxiety subscale of the AAS-R (r = 0.350, *p* < 0.01), and the Dyadic Satisfaction subscale of the DAS (r = −0.185, *p* < 0.05).

Moreover, the results showed some significant associations between SIP and important relationships, as shown in Table 4. Again, FoMO showed significant association with the cognitive social identity (r = 0.209, *p* < 0.01), the affective social identity (r = 0.224, *p* < 0.01), subjective (r = 0.225, *p* < 0.01) and group norms (r = 0.397, *p* < 0.01), and anxiety attachment (r = 0.502, *p* < 0.01).

We now turn to mediation and moderation analysis to examine in more detail the mechanisms through which anxious attachment influences IA, considering the mediating role of FoMO and the possible modulation of this effect by subjective and group norms. These two models allowed for the exploration of whether subjective norms or group norms (W) moderated the indirect impacts of adult anxious attachment (X) on IA (Y) through the mediation of FoMO (M) (Figure 1a,b).

Results showed that group norms moderated the effects of adult anxious attachment on FoMO (Coeff. = 0.070; *p* < 0.05; LLCI-ULCI: 0.0068; 0.1331). Specifically, the predictive effect of adult anxious attachment on FoMO was significant at all the levels of group norms (lower level: Effect = 0.2246, *p* < 0.001; medium level: Effect = 0.3120, *p* < 0.001; higher level: Effect = 0.4344, *p* < 0.001). However, the higher effect of adult anxious attachment on FoMO was at higher levels of group norms (Effect = 0.4344, *p* < 0.001). Conversely, group norms did not moderate the effect of FoMO on IA (Coeff. = 0.0490; *p* = 0.29; LLCI-ULCI: −0.0421; 0.1400) nor the effect of adult anxious attachment on IA (Coeff. = 0.0255; *p* = 0.56; LLCI-ULCI: −0.0618; 0.1129). Finally, the indirect effect of adult anxiety attachment on IA through the mediation of FoMO was significant at all levels of group norms (lower level: Effect = 0.1086; LLCI-ULCI: 0.0405; 0.2132; medium level: Effect = 0.1700; LLCI-ULCI: 0.1048; 0.2436; higher level: Effect = 0.2739; LLCI-ULCI: 0.1328; 0.3909).

About the possible moderating role of subjective norms, results showed that subjective norms moderated the effects of adult anxious attachment on FoMO (Coeff. = 0.0668; *p* < 0.05; LLCI-ULCI: 0.0116; 0.1221). Specifically, the predictive effect of adult anxious attachment on FoMO was significant at all the levels of subjective norms (lower level: Effect = 0.2456, *p* < 0.001; medium level: Effect = 0.3570, *p* < 0.001; higher level: Effect = 0.4684, *p* < 0.001). However, the higher effect of adult anxious attachment on FoMO was at higher levels of subjective norms (Effect = 0.4684, *p* < 0.001). Conversely, subjective norms did not moderate the effect of FoMO on IA (Coeff. = 0.0754; *p* = 0.11; LLCI-ULCI: −0.0174; 0.1681) nor the effect of adult anxious attachment on IA (Coeff. = 0.0040; *p* = 0.92; LLCI-ULCI: −0.0747; 0.0827). Finally, the indirect effect of adult anxiety attachment on IA through the mediation of FoMO was significant at all levels of subjective norms (lower level: Effect = 0.1214; LLCI-ULCI: 0.0412; 0.2344; medium level: Effect = 0.2213; LLCI-ULCI: 0.1417; 0.3018; higher level: Effect = 0.3492; LLCI-ULCI: 0.1981; 0.4797).

No significant effects emerged when age or marital status were considered as variables.

## 4. Discussion

The current study explored the roles of SIP (social identity, group norms, subjective norms), meaningful relationships (attachment, dyadic, and friendship relationships), and FoMO on IA among Italian young adults aged 18–30.

The first aim of our research was to explore the use of social networks, specifically Instagram, among young adults aged 18 to 30. Results showed that our sample of young adults, primarily women, who live with their families, work, study, or work-study, report using Instagram with an average daily use of 2.35 h. In terms of hours of Instagram use among young adults, our estimate was higher than the 1737 h reported by Soraci et al. (2022), while statistics show that the average time spent on Instagram in Italy is 2.43 h per week (Statista, 2024).

When we asked participants to describe their relationship with social networks, most of the benefits of using social networks became clear. However, some mentioned negative aspects like digital addiction, isolation, privacy concerns, and lack of control. Five main themes appeared in their open-ended responses about their relationship with social networks. One theme relates to relationships, such as meeting new people and maintaining existing ones. The second theme highlights the usefulness of social networks for searching for information or work. The third describes using social networks as a way to pass the time during downtime. The fourth focuses on digital addiction, and the fifth concerns privacy and control, such as checking what family, friends, and contacts are doing.

These findings are consistent with existing research and suggest that social networks offer significant opportunities across a range of areas, including information sharing, education, and marketing. By creating communities, social media connects people across physical distances and enables communication among those with similar interests. From a business perspective, social media can be used for advertising, direct communication, increasing brand awareness, and attracting new clients (Pekpazar et al., 2021). Furthermore, the development of friendships and romantic relationships may be facilitated by social networks, as they may prompt individuals to check their feeds more frequently, for example, waiting for a message or update from meaningful others. This facilitates exchange and communication (Soraci et al., 2022; Sharifi Fard et al., 2021). On the other hand, excessive use of social media can lead to typical behaviors relating to behavioral dependence (Longobardi et al., 2020).

The second aim was to explore the links between SIP (social identity, subjective norms, and group norms), important relationships (attachment, dyadic, and friendship), FoMO, and IA. Consistent with H1, the results showed significant links between most variables and IA. As supported by the literature, meaningful relationships (attachment, dyadic, and friendship) and FoMO were connected to IA (D’Arienzo et al., 2019; Rozgonjuk et al., 2020; Sun et al., 2020; Worsley et al., 2018). These patterns suggest that young adults who feel relational insecurity or higher FoMO might be more prone to loneliness or dissatisfaction, possibly turning to Instagram for connection or comfort. Additionally, SIP (social identity, subjective norms, and group norms) was positively related to IA, as previously shown (Marino et al., 2016). To understand these links, we can consider that problematic Instagram use among young adults is influenced by the people they care about (de Oliveira et al., 2016). Regarding how the variables affect IA, we saw that FoMO, adherence to social and group norms, and the level of group identification are all important factors.

The last goal was to examine how subjective and group norms influence the indirect effects of anxious attachment on IA through FoMO. Consistent with H2, the results revealed a significant indirect effect of anxious attachment on IA mediated by FoMO, aligning with prior research on problematic social media use (Boustead & Flack, 2021; Liu & Ma, 2019). These results indicate that individuals who tend to boost their self-esteem by seeking closeness with others (e.g., those with heightened anxious attachment styles) may overuse Instagram due to strong concerns about missing out on meaningful social experiences. However, the most innovative finding of this study was that subjective and group norms moderated the link between anxious attachment and FoMO. Specifically, the influence of anxious attachment on FoMO was greater when subjective and group norms were higher. In line with H3, the higher the subjective and group norms, the stronger the impact of anxious attachment on FoMO. In other words, subjective and group norms could intensify the negative effect of anxious attachment on FoMO, which may subsequently increase IA.

Overall, considering both quantitative and qualitative findings, the quantitative results show that variables like FoMO, subjective and group norms, and social group identification significantly influence IA among young adults. Specifically, anxious attachment indirectly affects IA through FoMO, with social norms moderating this relationship. These findings are supported by the qualitative responses to open-ended questions, where many participants described using social networks during moments of emptiness and boredom, such as during work breaks or before going to sleep at night. This “filler” use seems to reflect FoMO dynamics and the need to maintain social belonging, as shown in the quantitative analyses.

Furthermore, emergent themes about the benefits of social relationships, such as the chance to meet new people and stay connected over distances, confirm the central role of social norms and group identity in how often and intensely Instagram is used. Conversely, the concerns expressed qualitatively about addiction and control clearly highlight the negative effects linked to high FoMO and anxious attachment scores in the quantitative data. The combination of quantitative and qualitative data, as planned in the mixed-methods research design, offered a more complete understanding of the phenomenon and maintained consistency across the results, thereby reinforcing the evidence, even though it remains descriptive.

This study adds to the knowledge of IA among young adults aged 18–30 years. To our knowledge, the existing literature has not specifically targeted this exact group, but instead focuses on adults aged 18 and older or university students in general. Research has looked at personality traits (Kircaburun & Griffiths, 2018; Fard et al., 2025), emotional fatigue and “Instastress” (Sanz-Blas et al., 2019), and the protective role of critical thinking in IA (Fabio & Iaconis, 2024). Additional research has investigated IA and depression among college students (D’Souza & Hemamalini, 2018), the mediating role of loneliness in the bidirectional relationship between IA and life satisfaction (Rogowska & Libera, 2022), and how individuals with higher social anxiety interact differently with Instagram (Lopez & Polletta, 2021).

The findings of this study should be viewed considering certain limitations. First, the sampling method may have introduced significant selection bias, as indicated by the highly unbalanced gender representation. Additionally, the study relied on a relatively small sample size. Moreover, there was a higher proportion of women in the sample, which can cause greater variability in the results. This same limitation regarding the predominance of female respondents was also noted by Sanz-Blas et al. (2019), likely because social networking sites increasingly have more female than male users. Nonetheless, since the sample is skewed toward more women, it is not possible to generalize the results due to this limitation as well. Future research should aim for larger, more balanced samples. Another limitation is the exclusion of individual-level variables, as the study focused only on social and relational factors. Specifically, we did not consider factors such as earned secure attachment, existing mental health diagnoses, prior addiction behaviors, or total hours spent on social media beyond Instagram-specific use, which could introduce potential biases. Future research should include these variables, along with self-esteem, personality traits, and psychopathological risk. An additional limitation is that there are no established cut-off scores in the literature to categorize individuals’ average daily Instagram usage as low, medium, or high risk for developing IA. Future studies could address this gap. The absence of established cut-offs limits the accuracy of classifying participants’ Instagram use severity and may influence the interpretation of data and potential intervention strategies. It would also be helpful for future research to identify these cut-offs based on sociodemographic factors, such as gender or age groups. In fact, having a reference value for our target group would have improved clinical assessment and intervention strategies. Furthermore, the study’s cross-sectional design cannot establish causality. The results should be interpreted carefully, considering the possibility of bidirectional effects. Specifically, the findings do not allow us to conclude that SIP, meaningful relationships, or FoMO directly cause IA, without considering the potential for reciprocal influences. Future longitudinal studies are needed to clarify the direction of these relationships.

Although these limitations exist, this study emphasizes the importance of combining information from validated quantitative tools with in-depth qualitative insights. Qualitatively, it was possible to describe how young adults use social networks. The fact that many users spend a lot of time on social networks, especially Instagram, for fun, work promotion, information seeking, or relationship building, is not surprising. However, by comparing the data with the average hours spent on Instagram, consistent with previous research, excessive time on the internet, social networks, and Instagram may indicate behavioral addiction (Sanz-Blas et al., 2019; Soraci et al., 2022). On one hand, a mixed-methods approach provided a quantitative overview of social network use—particularly Instagram—by Italian young adults aged 18 to 30 and explored the role of SIP and meaningful relationships in IA. On the other hand, narrative stimulation offers a deeper exploration and understanding of participants’ relationships with social networks. Another innovative aspect of this study is its focus on content (social networks) rather than the medium (devices) in the context of addiction. Moreover, to our knowledge, this is the first research to examine an Italian sample of young adults aged 18–30 who use Instagram and to consider SIP alongside meaningful relationship variables related to IA.

## 5. Conclusions

The study gained insights into IA among Italian young adults by examining social influence processes, meaningful relationships, and FoMO. The results indicated that FoMO, social and group norms, and group identification significantly contribute to IA. Notably, anxious attachment indirectly affected IA through FoMO, with subjective and group norms strengthening this connection. This highlights the complex interaction between individual relational vulnerabilities and social factors. This new finding emphasizes the role of social norms in intensifying anxieties that lead to problematic Instagram use.

The mixed-methods approach allowed not only a quantitative evaluation of associations but also a qualitative understanding of young adults’ nuanced relationships with social networks, shedding light on both benefits and potential risks of usage. This holistic perspective advances the literature by integrating content-focused addiction factors within a social and relational framework for this specific age group.

Future research should address current limitations by employing larger, more balanced samples and longitudinal designs to clarify causal pathways. Expanding the model to include individual psychological traits, such as self-esteem and personality, and establishing cut-off points for IA severity would deepen understanding and improve the identification of at-risk individuals. Additionally, further qualitative studies could enrich interpretations of social media use dynamics. Overall, these findings provide a foundation for preventive interventions targeting social norms and relational factors to mitigate Instagram addiction in young adults.

## Figures and Tables

**Table 1:** Sociodemographic characteristics.

Total Sample (N = 180)	N (%)
Age in years, mean ± SD	M = 24.7 (SD = 3.25)
Sex at birth/current gender (same results) *	
Male	41 (22.8)
Female	136 (75.6)
Other	1 (0.6)
Not answer	2 (1.1)
Education	
Secondary school certificate	2 (1.1)
High school diploma	105 (58.3)
Bachelor’s degree	38 (21.1)
Master’s degree	31 (17.2)
Other	4 (2.2)
Marital status	
Married/Cohabitant	43 (23.9)
Separated/Divorced/Widowed	3 (1.7)
Single	134 (74.4)
Region of residence	
North	111 (61.6)
Centre	21 (11.7)
South & The Islands	48 (26.7)
Average annual income	
Up to 10 thousand euros	44 (24.4)
From 10 to 17 thousand euros	37 (20.6)
From 17 to 25 thousand euros	33 (18.3)
From 25 to 35 thousand euros	29 (16.1)
From 35 to 55 thousand euros	20 (11.1)
Over 55 thousand euros	17 (9.4)
Actual occupation	
Employed, self-employed	50 (27.8)
Worker, home worker, cooperative member	4 (2.2)
In the armed forces and law enforcement sector	1 (0.6)
Unemployed	8 (4.4)
University student	52 (28.9)
Student worker	51 (28.3)
Other	14 (7.8)
Who they live with	
Family of origin	108 (60.0)
Partner/Husband-wife	46 (25.6)
Housemate	9 (5.0)
Alone	15 (8.3)
Other	2 (1.1)

* We asked participants to report both their sex assigned at birth and their current gender identity, and the responses were consistent (i.e., sex assigned at birth matched current gender identity for all participants).

**Table 2:** Social Network and Instagram.

*Questions*	N (%)
Which Social Network are you registered on (besides Instagram)? (Multiple choice)	
WhatsApp	176 (97.8)
Facebook	156 (86.7)
YouTube	120 (66.7)
TikTok	102 (56.7)
Pinterest	75 (41.7)
LinkedIn	72 (40.0)
Twitter	53 (29.4)
BeReal	44 (24.4)
Snapchat	29 (16.1)
Other (Telegram)	4 (2.2)
Which Social Network do you use most often?	
Instagram	110 (61.6)
WhatsApp	39 (21.7)
TikTok	21(11.7)
Facebook	7 (3.9)
Twitter	2 (1.1)
YouTube	1 (0.6)
Do you ever lose hours of sleep because you stay up late on social media?	
Sometimes	65 (36.1)
Rarely	47 (26.1)
Never	37 (20.6)
Often	25 (13.9)
Always	6 (3.3)
When do you typically use Instagram during the day?	
Evening	71 (39.4)
Always	51 (28.3)
Afternoon	32 (17.8)
Something else	12 (6.7)
Morning	9 (5.0)
Night	5 (2.8)

**Table 3:** Themes and subthemes identified from participants’ narratives with related quotations.

Theme	Subtheme	Examples of Participants’ Quotations	(N%) *
BENEFITS			
1. Relationship	1. Meeting new people and making new connections	“A social changed my life because I met my husband, and let’s say my relationship with social is more of a way to spend some time or meet new people”	
	2. Keeping in touch with friends and family	“I find it a good way to maintain long-distance relationships with those you cannot see and hear from all the time and social media is a good way to shorten distances with both friends and relatives”	15 (25.0)
2. Utility	3. Searching for information (current affairs, travel, recipes, music, products and brands, videos)	“I can stay up-to-date on what is happening in the world and around me in terms of news and current affairs’ and ‘I mainly use them to organize trips, find places to visit, and particular restaurants.”	26 (43.3)
	4. Work	“I live on social, I am a content-creator, and I am a very active guy on social, I manage a page on Instagram and it goes very well”	
3. Filler for empty	5. During work breaks	“I use them mainly out of boredom and to occupy my time”	
	6. Morning or evening	“In the evening before falling asleep” and “I use social networks in the morning or during breaks at work”	10 (16.7)
COSTS			
1. Addiction	7. Spending a lot of time on social networks to the detriment of our passions and human relationships	“Social media takes up a lot of time that I could be spending on my studies and my passions. I think I’m addicted to them.”	5 (8.3)
2. Privacy & Control	9. Checking what family, friends, contacts are doing	“I realise I have tics. Now and then I open Instagram, check one/two stories to see what others are doing and close it”	4 (6.6))

* Frequency of responses (N%), total sample N = 60.

**Table 4:** Correlational Analysis. * *p* ˂ 0.05; ** *p* ˂ 0.01.

	1	2	3	4	5	6	7	8	9	10	11
1. Instagram Addiction (IAS)	1										
2. Social Identity (Cognitive)	0.172 *	1									
3. Social Identity (Affective)	0.064	0.546 **	1								
4. Social Identity (Evaluative)	−0.084	0.492 **	0.723 **	1							
5. Subjective Norms	0.231 **	0.138	0.063	0.018	1						
6. Group Norms	0.433 **	0.102	0.011	−0.089	0.670 **	1					
7. Fear of Missing Out (FoMOs)	0.623 **	0.209 **	0.224 **	0.017	0.225 **	0.397 **	1				
8. Adult Anxiety Attachment (AAS-R)	0.350 **	0.050	0.047	−0.222 **	0.070	0.176 *	0.502 **	1			
9. Dyadic Satisfaction (DAS)	−0.185 *	0.267 **	0.247 **	0.378 **	0.050	−0.177 *	−0.148	−0.223 *	1		
10. Friendship Quality Scale—Security (FQS)	−0.267 **	0.235 **	0.223 **	0.311 **	−0.168 *	−0.222 **	−0.159	−0.055	0.345 **	1	
11. Friendship Quality Scale—Closeness (FQS)	−0.097	0.153	0.335 **	0.253 **	−0.014	−0.034	−0.002	0.105	0.359 **	0.543 **	1

## Data Availability

The data that support the findings of this study are available from the corresponding author upon reasonable request. Due to privacy and ethical restrictions related to the sensitive nature of the research, the data are not publicly available.
